# Caspase-8 Regulates Endoplasmic Reticulum Stress-Induced Necroptosis Independent of the Apoptosis Pathway in Auditory Cells

**DOI:** 10.3390/ijms20235896

**Published:** 2019-11-24

**Authors:** Akihiro Kishino, Ken Hayashi, Miyoko Maeda, Toyoharu Jike, Chiaki Hidai, Yasuyuki Nomura, Takeshi Oshima

**Affiliations:** 1 Department of Otolaryngology, School of Medicine, Nihon University, 30-1, Oyaguchi Kami-cho, Itabashi-ku, Tokyo 173-8610, Japan; 2 Department of Otolaryngology, Kamio Memorial Hospital, Tokyo 101-0063, Japan; 3 Research Institute of Medical Science, School of Medicine, Nihon University, Tokyo 173-8610, Japan; 4 Department of Physiology, School of Medicine, Nihon University, Tokyo 173-8610, Japan

**Keywords:** endoplasmic reticulum stress, necroptosis, apoptosis, auditory cells

## Abstract

The aim of this study is to elucidate the detailed mechanism of endoplasmic reticulum (ER) stress-induced auditory cell death based on the function of the initiator caspases and molecular complex of necroptosis. Here, we demonstrated that ER stress initiates not only caspase-9-dependent intrinsic apoptosis along with caspase-3, but also receptor-interacting serine/threonine kinase (RIPK)1-dependent necroptosis in auditory cells. We observed the ultrastructural characteristics of both apoptosis and necroptosis in tunicamycin-treated cells under transmission electron microscopy (TEM). We demonstrated that ER stress-induced necroptosis was dependent on the induction of RIPK1, negatively regulated by caspase-8 in auditory cells. Our data suggested that ER stress-induced intrinsic apoptosis depends on the induction of caspase-9 along with caspase-3 in auditory cells. The results of this study reveal that necroptosis could exist for the alternative backup cell death route of apoptosis in auditory cells under ER stress. Interestingly, our data results in a surge in the recognition that therapies aimed at the inner ear protection effect by caspase inhibitors like zVAD-fmk might arrest apoptosis but can also have the unanticipated effect of promoting necroptosis. Thus, RIPK1-dependent necroptosis would be a new therapeutic target for the treatment of sensorineural hearing loss due to ER stress.

## 1. Introduction

The endoplasmic reticulum (ER) is the central cellular organelle responsible for synthesis and maturation of transmembrane and secretory proteins [1,2]. The ER stress is the intracellular stress caused by the accumulation of unfolded or misfolded proteins in the ER lumen [3]. Under ER stress, unfolded protein response (UPR), the adaptive system conserved from yeast to eukaryotic cells, is activated to restore ER homeostasis [4]. However, sustained ER stress ultimately leads to cell death [5]. Accumulating evidence suggests that ER stress-induced cellular dysfunction and death is involved in the pathogenesis of several human diseases, such as neurodegenerative diseases, psychiatric diseases, and aging [6,7,8]. Furthermore, it has been reported that ER stress results in acute sensorineural hearing loss or age-related hearing loss, inducing apoptosis in auditory cells [9,10,11]. However, it is clinically difficult to attribute the hearing loss to apoptosis (programmed cell death) because the hearing level varies widely in the treatment process.

Historically, cell death has been divided into three types based on morphological criteria; type I (apoptosis), type II (autophagic cell death), and type III (necrosis) [12,13]. It has been widely reported that ER stress induces apoptosis, which relies on the activation of caspase cascades [14]. Specific morphological characteristics of apoptotic cells are cell shrinkage, chromatin condensation, and nucleus fragmentation [13]. The morphologic features of apoptosis result from the activation of caspases, like caspase-3 [15]. Tunicamycin is one of the widely used ER stress inducers [16]. It inhibits N-linked glycosylation of immature proteins [17], which leads to the accumulation of misfolded or unfolded proteins in the ER and ultimately results in cell death [18]. Some reports have demonstrated that the treatment of tunicamycin-induced apoptosis is characterized by the expression of C/EBP homologous protein (CHOP), a specific ER stress-associated pro-apoptotic factor, in hair cells and spinal ganglion cells in the cochlea [9,10]. The ER stress-induced apoptosis in hair cells and spinal ganglion cells caused sensorineural hearing loss [9,10,11]. Recently, it has been appreciated that various types of non-apoptotic cell deaths, like necroptosis, exist [19]. Necroptosis is a programmed necrotic cell death, defined as a distinct form of cell death that is caspase-independent and mediated through the formation of the receptor-interacting serine/threonine kinase (RIPK)1/RIPK3 complex called necrosome. Necroptosis is involved in many pathological processes of human diseases, such as ischemic brain injury, injury-induced inflammatory, and neurodegenerative diseases [20,21,22]. Mixed lineage kinase domain-like protein (MLKL) is a key downstream factor of RIPK3 in the necroptosis pathway. During RIPK3 activation, MLKL is translocated to the plasma membrane where it causes membrane lysis, which eventually results in necroptosis [23,24,25,26]. A report has demonstrated that there is the crosstalk between apoptosis and necroptosis through caspase-8 [27]. Specifically, apoptosis inhibits necroptosis by RIPK1 cleavage through the activation of caspase-8 [28]. This indicates that caspase-8 functions as an important negative regulator of RIPK1. However, the crosstalk between apoptosis and necroptosis through caspase-8 and RIPK1 in auditory cells remains unclear.

Recently, several reports have suggested that ER stress causes necroptosis [29,30,31,32]. The induction of ER stress by tunicamycin caused necroptosis in fibroblasts has also been indicated [33,34]. Endoplasmic reticulum stress is widely reported to induce caspase-dependent apoptosis through an intrinsic pathway [14]. Apoptosis occurs through two main pathways, extrinsic and intrinsic pathway. The extrinsic pathway originates from the activation of cell-surface death receptors and results in the activation of initiator caspase-8. The intrinsic pathway originates from the mitochondrial release of cytochrome c and associated activation of initiator caspase-9 [35]. The initiator caspases, caspase-8 and caspase-9, cleave inactive forms of effector caspase-3, thereby activating them. Effector caspase-3, in turn, cleave other protein substrates in the cell, resulting in the apoptotic process [14,36]. However, the function of the initiator caspases on ER stress-induced apoptosis and necroptosis has not been elucidated in auditory cells.

We have reported on the crosstalk between the ER stress signaling pathway and autophagy in auditory cells [37]. However, the detailed mechanism of ER stress-induced auditory cell death based on the function of the initiator caspases and molecular complex of necroptosis remained unclear. The investigation of the mechanisms of ER stress-induced cell death in auditory cells focusing on the association of necroptosis and apoptosis can make a major contribution to the understanding of the pathogenesis of inner ear diseases, including sensorineural hearing loss. Furthermore, understanding the precise mechanisms of ER stress-induced cell death in auditory cells could provide significant therapeutic insights and lead to the development of novel therapeutics. Therefore, we investigated the mechanism of ER stress-induced cell death in auditory cells.

## 2. Results

### 2.1. ER Stress Induces Not Only Apoptosis but also Necroptosis in HEI-OC1 Cells

**Figure 1:**
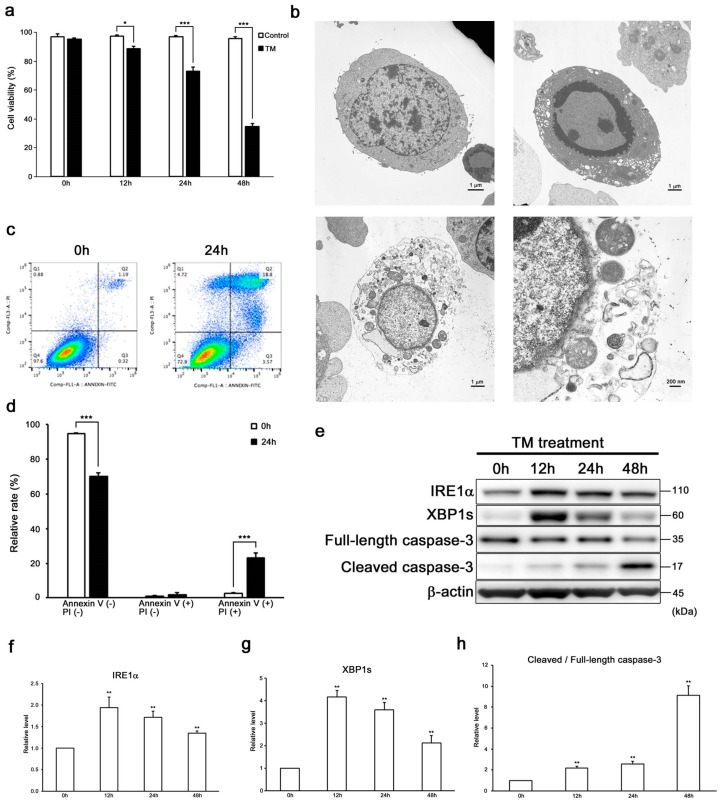
Endoplasmic reticulum (ER) stress induces apoptosis in HEI-OC1 cells. (**a**) Cell viability was decreased in a time-dependent manner in 50 µg/mL tunicamycin-treated HEI-OC1 cells. The data are represented as means ± S.D. of three or more independent studies (* *p* < 0.05 and *** *p* < 0.001 compared to the control group, determined using unpaired Student’s *t*-test). (**b**) Representative transmission electron microscopy photomicrographs of HEI-OC1 cells treated with tunicamycin (50 µg/mL for 24 h). The control cells showed normal cell organelles and nucleus. (upper left panel, 5000× magnification). Apoptotic cells showed condensed cytosol and marginalized chromatin and nuclear fragmentation (upper right panel, 5000× magnification). Necroptotic cells showed disrupted plasma membrane, translucent cytoplasm, swelled organelles, and preserved nuclear membrane (lower panel, left: 5000× and right: 20,000× magnifications). Transmission electron microscopy images of low magnifying scales are presented in Figure S1a. (**c**,**d**) Flow cytometry analysis showed that tunicamycin treatment (50 µg/mL for 24 h) increased the populations of late apoptotic and necrotic cells (Annexin V+, PI+). The data are shown as means ± S.D. of three or more independent studies (*** *p* < 0.001 compared to the 0 h group, determined using unpaired Student’s *t*-test). (**e**–**h**) Representative Western blots showing the expressions of IRE1-α, XBP1s, and cleaved/full-length caspase-3 in tunicamycin-treated cells (50 µg/mL for 48 h). β-actin was included as the loading control. The expressions of IRE1-α, XBP1s, and cleaved/full length caspase-3 were detected, and the means ± S.D. (fold changes compared to the control group) of three or more independent studies were presented (** *p* < 0.01 compared to the 0 h group, determined using one-way ANOVA followed by Bonferroni test). Full-length blots are presented in Figure S1b–f.

Sustained ER stress induces auditory cell death (Figure 1a) [37]. To detect the cell death modality induced by ER stress, first we observed the morphology of tunicamycin-treated cells by transmission electron microscopy (TEM) (Figure 1b and Figure S1a). Transmission electron microscopy revealed that cells in the control condition exhibited normal nuclei and organelles. In contrast, cells treated with tunicamycin displayed condensed cytosol and marginalized chromatin and nuclear fragmentations, which were typical morphological characteristics of apoptosis. On the other hand, some tunicamycin-treated auditory cells showed a disrupted plasma membrane, translucent cytoplasm, swollen mitochondria, and preserved nuclear membrane. These morphological changes indicated characteristics of necroptosis [21,38,39].

Then, we performed flow cytometry analysis and evaluated the expression of cleaved/full-length caspase-3 by Western blot analysis to clarify the differences between apoptosis and necroptosis (Figure 1c–h). Indeed, flow cytometry analysis also showed that tunicamycin treatment induced the increase in populations of both late apoptotic and necrotic cells. Western blot analysis revealed increased expression levels of the ER stress marker inositol-requiring protein1α (IRE1α) and spliced X-box-binding protein 1 (XBP1s), and the apoptosis marker cleaved/full-length caspase-3 in tunicamycin-treated cells. These results suggested ER stress induced apoptosis in auditory cells.

**Figure 2:**
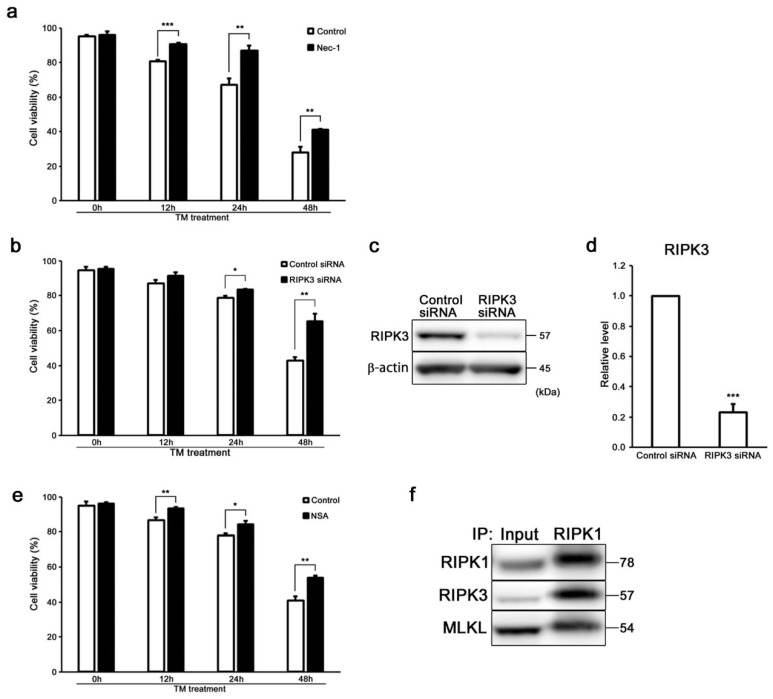
ER stress induces necroptosis in HEI-OC1 cells. (**a**) After Nec-1 treatment (20 µM for 24 h), the cells were treated with tunicamycin (50 µg/mL for 48 h), and cell viability was determined by trypan blue staining. The data are represented as means ± S.D. of three or more independent studies (** *p* < 0.01 and *** *p* < 0.001 compared to the control group, determined using unpaired Student’s *t*-test). (**b**) After transfection with RIPK3 and control siRNA for 48 h, the cells were treated with tunicamycin (50 µg/mL for 48 h), and cell viability was determined by trypan blue staining. The data are represented as means ± S.D. of three or more independent studies (* *p* < 0.05 and ** *p* < 0.01 compared to the control group, determined using unpaired Student’s *t*-test). (**c**,**d**) Representative Western blots showing the expression of RIPK3 in RIPK3 KD cells. β-actin was included as the loading control. The expression of RIPK3 was detected, and the means ± S.D. (fold changes compared to the control group) of three or more independent studies were presented (*** *p* < 0.001 compared to the control group, determined using unpaired Student’s *t*-test). Full-length blots are presented in Figure S2a,b. (**e**) After NSA treatment (2.5 µM for 24 h), the cells were treated with tunicamycin (50 µg/mL for 48 h), and cell viability was determined by trypan blue staining. The data are represented as means ± S.D. of three or more independent studies (* *p* < 0.05 and ** *p* < 0.01 compared to the control group, determined using unpaired Student’s *t*-test). (**f**) Co-immunoprecipitation revealed physical interactions between RIPK1, RIPK3, and MLKL in tunicamycin-treated cells (50 µg/mL for 24 h). Data presented are representative of three independent experiments. Full-length blots are presented in Figure S2c–e.

On the basis of these findings, we hypothesized that ER stress could induce not only apoptosis, but also necroptosis in auditory cells. In order to investigate whether ER stress by tunicamycin induces necroptosis in auditory cells after pretreatment with necrostatin-1 (Nec-1), a RIPK1 allosteric inhibitor, cells were treated with tunicamycin and then the cell viability was measured. As shown in Figure 2a, the cell viability in the cells treated with tunicamycin, in combination with Nec-1, significantly increased more than that of the cells treated with tunicamycin alone. Next, we knocked down (KD) RIPK3 using small interfering RNA (siRNA) and then evaluated the cell viability (Figure 2b–d). Tunicamycin-treated RIPK3 KD cells showed a significant increase in cell viability compared with tunicamycin-treated si-control cells. It has been reported that MLKL is a key molecule mediating necroptosis downstream of RIPK3 [23,24,25,26]. In order to investigate whether MLKL is involved in the necroptosis signaling pathway in auditory cells, after pretreatment with necrosulfonamide (NSA), an MLKL allosteric inhibitor, cells were treated with tunicamycin, and then the cell viability was measured. As shown in Figure 2e, the viability of the cells treated with tunicamycin, in combination with NSA, significantly increased more than that of the cells treated with tunicamycin alone. Next, we performed a co-immunoprecipitation assay to detect the direct interaction between RIPK1, RIPK3, and MLKL. Co-immunoprecipitation revealed that physical interactions between RIPK1, RIPK3, and MLKL in tunicamycin-treated cells (Figure 2f). These results suggested that MLKL was involved in ER stress-induced necroptosis signaling pathway in auditory cells. Taken together, these results suggested that ER stress induced not only apoptosis, but also necroptosis in auditory cells.

### 2.2. ER Stress Induces RIPK1-Dependent Necroptosis in HEI-OC1 Cells

**Figure 3:**
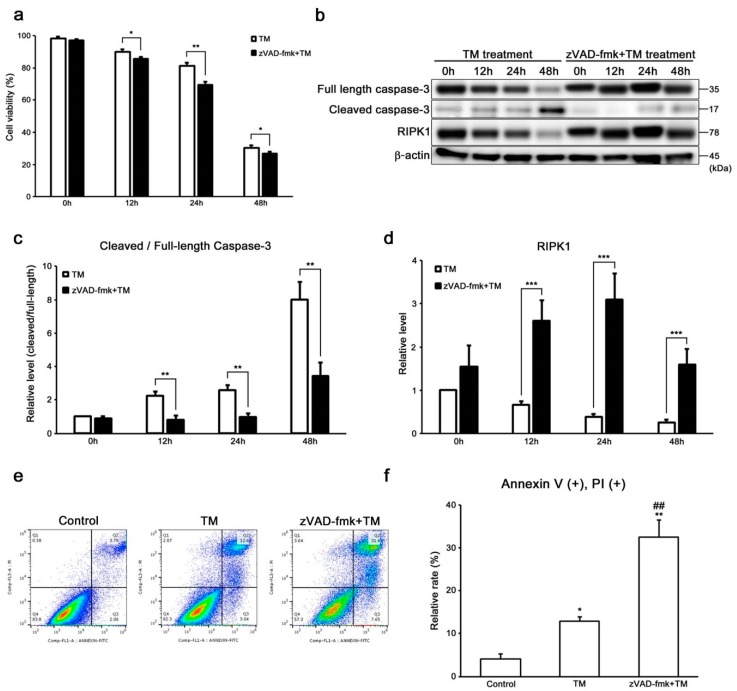
ER stress induces RIPK1-dependent necroptosis in HEI-OC1 cells. (**a**) After treatment with zVAD-fmk (20 µM for 5 h), the cells were treated with tunicamycin (50 µg/mL for 48 h), and cell viability was determined by trypan blue staining. The data are represented as means ± S.D. of three or more independent studies (* *p* < 0.05 and ** p < 0.01 compared to the control group, determined using unpaired Student’s *t*-test). (**b**–**d**) Representative Western blots showing the expressions of the cleaved-/full-length caspase-3 and RIPK1 in zVAD-fmk-treated cells after tunicamycin treatment (50 µg/mL for 48 h). β-actin was included as the loading control. The expressions of cleaved/full-length caspase-3 and RIPK1 were detected, and the means ± S.D. (fold changes compared to the control group) of three or more independent studies were presented (** *p* < 0.01 and *** *p* < 0.001 compared to the control group, determined using unpaired Student’s *t*-test). Full-length blots are presented in Figure S3a–d. (**e**,**f**) Flow cytometry analysis showed an increase in the populations of late apoptotic and necrotic cells (Annexin V+, PI+) in zVAD-fmk-treated cells after tunicamycin treatment (50 µg/mL for 24 h). The data are shown as means ± S.D. of three or more independent studies (* *p* < 0.05 and ** *p* < 0.01 compared to the control group, ## *p* < 0.01 compared to the tunicamycin-treated group, determined using one-way ANOVA followed by Bonferroni test).

We investigated whether ER stress induces either caspase-dependent or -independent cell death using Z-Val-Ala-Asp-fluoromethylketone (zVAD-fmk), a pan-caspase inhibitor before tunicamycin treatment for clarifying differences between apoptosis and necroptosis in auditory cells. Surprisingly, the cell viability was significantly decreased, although the expression level of cleaved/full-length caspase-3 was significantly suppressed in zVAD-fmk-treated cells (Figure 3a–c). These results suggest that caspase-3-independent pathway exists in auditory cell death induced by ER stress. We then evaluated the expression of RIPK1 in zVAD-fmk-treated cells by Western blot analysis to uncover the function of RIPK1 as a mediator of necroptosis in auditory cells under ER stress. Importantly, the expression level of RIPK1 was significantly increased in zVAD-fmk-treated cells (Figure 3b,d). Flow cytometry analysis showed a significant increase in the population of both late apoptotic and necrotic cells in the cells treated with tunicamycin in combination with zVAD-fmk compared with the cells treated with tunicamycin alone (Figure 3e,f). Taken together, these results suggested that ER stress-induced necroptosis might be dependent on RIPK1 in auditory cells.

### 2.3. Caspase-8 Regulates ER Stress-Induced Necroptosis in HEI-OC1 Cells

**Figure 4:**
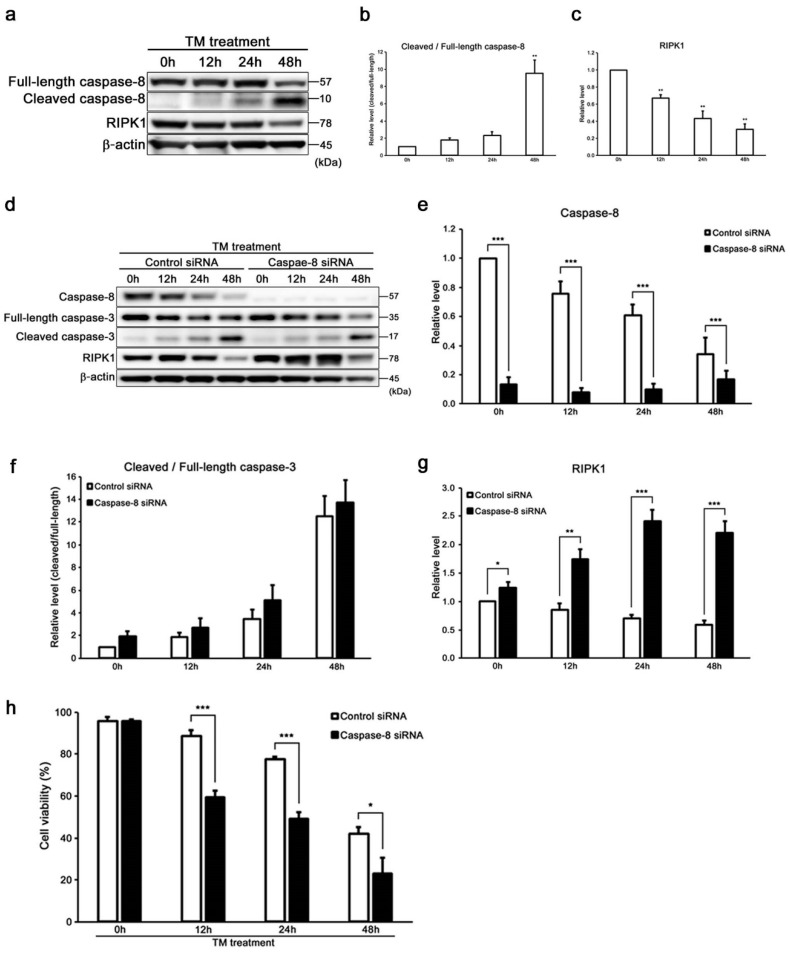
Caspase-8 regulates ER stress-induced necroptosis in HEI-OC1 cells. (**a**–**c**) Representative Western blots showing the expressions of cleaved/full-length caspase-8 and RIPK1. β-actin was included as the loading control. The expressions of cleaved/full-length caspase-8 and RIPK1 were detected, and the means ± S.D. (fold changes compared to the control group) of three or more independent studies were presented (** *p* < 0.01 compared to the 0 h group, determined using one-way ANOVA followed by Bonferroni test). Full-length blots are presented in Figure S4a–d. (**d**–**g**) Representative Western blots showing the expressions of caspase-8, cleaved/full-length caspase-3, and RIPK1 in tunicamycin-treated caspase-8 KD cells (50 µg/mL for 48 h). Knockdown of caspase-8 upregulated the expression level of RIPK1. β-actin was included as the loading control. The expressions of caspase-8, cleaved/full-length caspase-3, and RIPK1 were detected, and the means ± S.D. (fold changes compared to the control group) of three or more independent studies were presented (* *p* < 0.05, ** *p* < 0.01, and *** *p* < 0.001 compared to the control group, determined using unpaired Student’s *t*-test). Full-length blots are presented in Figure S4e–i. (**h**) After transfection with caspase-8 and control siRNA for 48 h, the cells were treated with tunicamycin (50 µg/mL for 48 h), and cell viability was determined by trypan blue staining. The data are represented as means ± S.D. of three or more independent studies (* *p* < 0.05 and *** *p* < 0.001 compared to the control group, determined using unpaired Student’s *t*-test).

Recently, it was reported that RIPK1 is negatively regulated by caspase-8 [27,28]. This suggests that caspase-8 might be a key regulator, making a distinction between apoptosis and necroptosis. We evaluated the expressions of cleaved/full-length caspase-8 and RIPK1 in tunicamycin-treated cells to study the cell death signaling pathway based on apoptosis and necroptosis in auditory cells (Figure 4a). The increase in the expression of cleaved/full-length caspase-8 was time-dependent in the tunicamycin-treated cells (Figure 4b). On the other hand, the expression of RIPK1 was decreased in a time-dependent manner (Figure 4c). These findings showed that there was a significant inverse correlation between cleaved/full-length caspase-8 and RIPK1 expression. Then, the expression of RIPK1 was evaluated in tunicamycin-treated caspase-8 KD cells (Figure 4d–g). The expression of RIPK1 was significantly up regulated in caspase-8 KD cells. However, no significant difference was observed between si-control and caspase-8 KD cells in the expression level of cleaved/full-length caspase-3. Surprisingly, the cell viability was decreased in tunicamycin-treated caspase-8 KD cells than in si-control cells (Figure 4h). These results suggested that caspase-8 suppresses ER stress-induced necroptosis through negatively regulating RIPK1 in auditory cells, whereas it did not affect caspase-3-dependent apoptosis.

### 2.4. Caspase-9 Influences the Induction of Intrinsic Apoptosis with Caspase-3 in HEI-OC1 Cells

**Figure 5:**
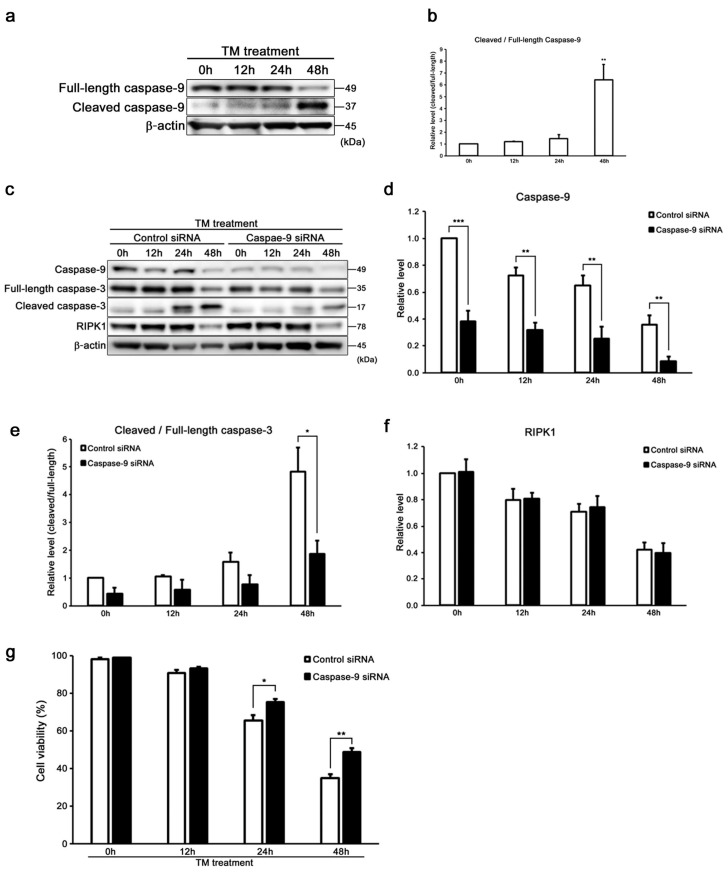
Caspase-9 influenced the induction of intrinsic apoptosis along with caspase-3 in HEI-OC1 cells. (**a**,**b**) Representative Western blots showing the expression of cleaved/full-length caspase-9. β-actin was included as the loading control. The expression of cleaved/full-length caspase-9 was detected, and the means ± S.D. (fold changes compared to the control group) of three or more independent studies were presented (** *p* < 0.01 compared to the 0 h group, determined using one-way ANOVA followed by Bonferroni test). Full-length blots are presented in Figure S5a–c. (**c**–**f**) Representative Western blots showing the expressions of caspase-9, cleaved/full-length caspase-3, and RIPK1 in tunicamycin-treated caspase-9 KD cells (50 µg/mL for 48 h). β-actin was included as the loading control. The expressions of caspase-9, cleaved/full-length caspase-3, and RIPK1 were detected, and the means ± S.D. (fold changes compared to the control group) of three or more independent studies were presented (* *p* < 0.05, ** *p* < 0.01, and *** *p* < 0.001 compared to the control group, determined using unpaired Student’s *t*-test). Full-length blots are presented in Figure S5d–h. (**g**) After transfection with caspase-9 and control siRNA for 48 h, the cells were treated with tunicamycin (50 µg/mL for 48 h), and cell viability was determined by trypan blue staining. The data are represented as means ± S.D. of three or more independent studies (* *p* < 0.05 and ** *p* < 0.01 compared to the control group, determined using unpaired Student’s *t*-test).

It was reported that caspase-9 and caspase-3 have distinct roles during intrinsic apoptosis [14,40]. The expression of cleaved/full-length caspase-9 was increased time-dependently in tunicamycin-treated cells (Figure 5a,b). Then, we confirmed the expression of cleaved/full-length caspase-3 in tunicamycin-treated caspase-9 KD cells to investigate whether ER stress by tunicamycin induces intrinsic apoptosis in auditory cells. As shown in Figure 5c–e, tunicamycin-treated caspase-9 KD cells showed significantly suppressed cleaved/full-length caspase-3 expression level. The decreasing rate of cell viability was significantly suppressed in tunicamycin-treated caspase-9 KD cells than in si-control cells (Figure 5g). These results suggest that ER stress induced by tunicamycin initiates caspase-3 activation via the intrinsic apoptotic pathways in auditory cells. Next, we evaluated the expression of RIPK1 in caspase-9 KD cells by Western blot analysis to confirm the effect of caspase-9 to necroptosis in auditory cells under ER stress. As shown in Figure 5c,f, there is no significant difference in RIPK1 expression between si-control and caspase-9 KD cells. These results indicate that caspase-9 has no effect on the induction of necroptosis in auditory cells.

## 3. Discussion

**Figure 6:**
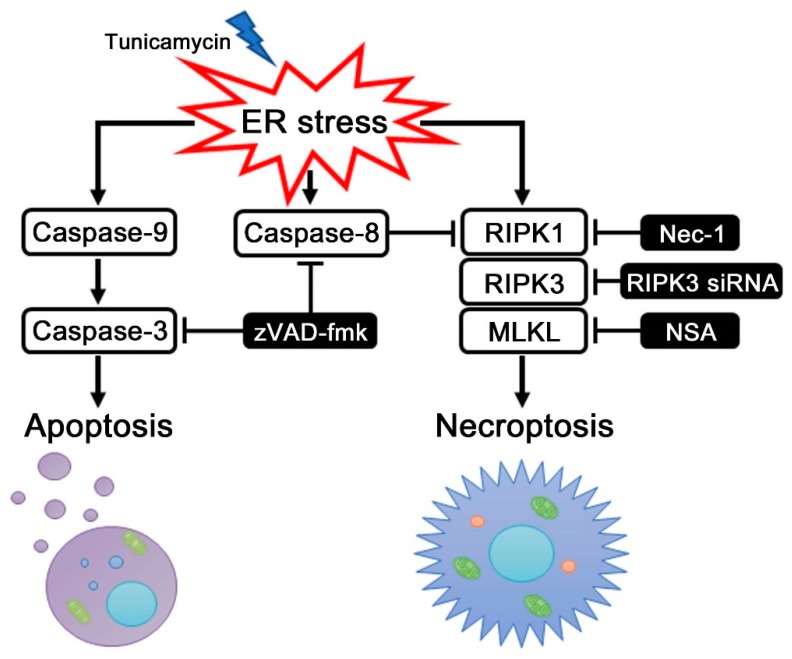
ER stress induces RIPK1-dependent necroptosis and caspase-9-dependent intrinsic apoptosis in auditory cells. A schematic model shows ER stress induces necroptosis and apoptosis in auditory cells. RIPK1-dependent necroptosis is regulated negatively by caspase-8 under ER stress. ER stress-induced intrinsic apoptosis depends on the induction of caspase-9 along with caspase-3.

In this study, we provided evidence that ER stress induced by tunicamycin initiates not only caspase-9-dependent intrinsic apoptosis along with caspase-3, but also RIPK1-dependent necroptosis in auditory cells. First, we demonstrated that ER stress-induced necroptosis was dependent on RIPK1 in auditory cells, and negatively regulated by caspase-8. Secondly, our data suggested that ER stress-induced intrinsic apoptosis depends on the induction of caspase-3 and caspase-9 in auditory cells. To our knowledge, this is the first report indicating that caspase-8 negatively regulates necroptosis via RIPK1, and caspase-9 activation initiates apoptosis along with caspase-3 in auditory cells under ER stress (Figure 6).

Sensorineural hearing loss is one of the most frequent physical disabilities in the world, and hearing impairment has an exceedingly bad impact on the person’s quality of life. A variety of research has been done for the hearing protection in basic principles and clinical applications. Some reports from the point of cell biology demonstrated that the unfolded protein response (UPR) triggered by endoplasmic reticulum (ER) stress is deeply involved in the pathogenesis of sensorineural hearing loss, inducing apoptosis in cochlear hair cells, stria vascularis and spiral ganglion cells [9,10,11,41,42,43,44]. It has been widely reported that ER stress induces apoptosis, which depends on the activation of the caspase cascade [14,40]. Recent studies have shown that ER stress causes not only apoptosis but also necroptosis [17,18,19,20,29,30,31,32,45]. Necroptosis is a form of caspase-independent cell death and is programmed necrosis relying on the activity of necrosome. We have previously reported that sustained ER stress by tunicamycin induces auditory cell death [37]. However, the detailed mechanism of ER stress-induced cell death in auditory cells has not been fully understood.

As an initial step to investigate the modality of ER stress-induced cell death, we observed the existence of two different findings for apoptosis and necroptosis in ER stress-treated auditory cells under TEM [13,15,21,38,39] (Figure 1b) and an increase of populations of the late apoptotic and necrotic cells in flowcytometry analysis (Figure 1c,d). These results indicate that ER stress could induce both apoptosis and necroptosis in auditory cells, in agreement with the previous reports [45].

Necroptosis is mediated through the formation of the RIPK1/RIPK3 complex called necrosome [29,30,31,32]. Necrosome is the key molecular component of the necroptosis signaling cascade [46,47]. The MLKL is a key downstream factor of RIPK3 in the necroptosis pathway and is a core component of RIPK1/RIPK3 necrosome [23,24,25,26,48]. Our results indicated that the cell viabilities were significantly increased in Nec-1 or NSA-treated cells and RIPK3 KD cells [48] (Figure 2a–e), and co-immunoprecipitation revealed the direct interaction between RIPK1, RIPK3, and MLKL (Figure 2f). These results suggest that ER stress induces necroptosis, stimulating the molecular complex of RIPK1, RIPK3, and MLKL in auditory cells.

Interestingly, we considered that it is not enough to regulate only apoptosis for protecting auditory cells from ER stress since the cell viability was significantly decreased in cells treated with tunicamycin in combination with zVAD-fmk, a pan-caspase inhibitor, although the expression of cleaved/full-length caspase-3 was suppressed (Figure 3a–c), and the expression of RIPK1 was significantly upregulated in the zVAD-fmk-treated cells. Necroptosis is reported to be enhanced by zVAD-fmk [28,49] The morphological characteristics of necroptotic cells are disrupted plasma membrane, translucent cytoplasm, and preserved nuclear membrane [21,38,39]. PI cannot cross intact plasma membrane and therefore will only be present in DNA of cells where the plasma membrane has been permeabilized. Based on this theory, our flow cytometry data suggests that zVAD-fmk treatment increases PI positive and Annexin V positive cells showing usually late apoptosis and necrosis with disrupted plasma membrane (Figure 3e,f). However, the formation of small pores in the plasma membrane of cells can be considered as a general feature in necroptosis that drives final membrane disruption, promoting PI intake [50]. In addition, the expression of RIPK1 was extremely increased in TM and zVAD-fmk-treated cells. These results indicated that necroptosis could be regulated by the induction of RIPK1. Taken together, these data suggest that targeting apoptosis based on caspase-3 for suppressing auditory cell death caused by ER stress is not sufficient, and it is necessary to regulate not only apoptosis but also necroptosis for protecting auditory cell from ER stress.

Caspase-8 was reported to suppress necroptosis through inactivating RIPK1 by cleavage, in addition to activating apoptosis [27,28]. Thus, necroptosis serves as an alternative cell death route of apoptosis. Here, we focused on the regulation of both apoptosis and necroptosis in auditory cells by caspase-8. The expressions of cleaved/full-length caspase-8 and RIPK1 showed an inverse correlation (Figure 4a–c). Based on these findings, we identified that caspase-8 KD cells showed a significant increase in the expression of RIPK1 and a significant decrease in cell viability and in the population of zVAD-fmk-treated cells (Figure 3a,b,d and Figure 4d,e,g,h). However, the expression of cleaved/full-length caspase-3 and MLKL did not change in caspase-8 KD cells compared to the si-control cells (Figure 4d,f and Figure S4j). These results indicated that RIPK1, not MLKL, should be a key regulator of necroptosis induction in auditory cells under tunicamycin-induced ER stress. On the other hand, caspase-9 has an important role in the intrinsic apoptotic pathway in auditory cells under tunicamycin-induced ER stress, and in interacting with caspase-3 (Figure 5c–g). Our study suggests that caspase-8 activation inhibits ER stress-induced necroptosis through the negative regulation of RIPK1 expression independently of the apoptosis pathway in auditory cells under ER stress. Necroptosis is involved in the pathological process of various diseases, such as ischemic brain injury, injury-induced inflammatory disease, and neurodegenerative diseases [20,21,22]. The induction of necroptosis provokes strong inflammatory responses, which shows a detrimental effect on various organs [51,52]. As shown in Figure 3a and Figure 4h, necroptosis enhancement resulted in an increase in auditory cell death. This result suggests that necroptosis could exist as an alternative cell death route of apoptosis in auditory cells under ER stress. Based on these findings, we speculated that necroptosis is considered an important backup mechanism of apoptosis, and caspase-8 inhibits necroptosis by suppressing the expression of RIPK1 through the independent pathway to apoptosis under ER stress in auditory cells. This result suggested that caspase inhibitors, like zVAD-fmk, have two sides of arresting apoptosis and promoting necroptosis. Namely, our results indicated that a new treatment targeting on necroptosis should be established for protecting inner ear from ER stress. ER stress was reported to be involved in cochlear cell apoptosis in a cisplatin-induced ototoxicity rat model, inducing sensorineural hearing loss [42]. Recent studies reported that cisplatin and aminoglycoside ototoxicity induce necroptosis and apoptosis in organ of Corti and spiral ganglion cells, which lead to sensorineural hearing loss in in vivo models [53,54]. However, no study has demonstrated crosstalk between caspases and necrosome in apoptosis and necroptosis under ER stress. In this study, we investigated the precise mechanism of ER stress-induced auditory cell death by focusing on the function of the initiator caspases and molecular complex of necroptosis and showed that ER stress induced both apoptosis and necroptosis in auditory cells, based on the previous study that ER stress inhibitor attenuates auditory cell death both in vitro and in vivo [55,56].

ER stress has been reported to be associated with the pathogenesis of hereditary hearing loss such as Wolfram syndrome (WFS) [43]. Increasing vulnerability against ER stress by wolframin protein deficiency contributes to the pathogenesis of WFS. WFS-deficient β-cells are susceptible to ER stress-mediated apoptosis [43,57]. However, there are currently no studies that have reported the involvement of ER stress-induced necroptosis in the pathogenesis of WFS. Our study should be a new insight for understanding the pathogenesis of WFS.

In conclusion, our study reveals that ER stress induces not only caspase-9 dependent apoptosis along with caspase-3, but also RIPK1-dependent necroptosis in auditory cells. In this process, caspase-8 regulates necroptosis despite not being involved in caspase-3 activation. To our knowledge, this is the first ever study to show that ER stress induced both apoptosis and necroptosis in auditory cells. The study also contributed to pathology underlying hearing impairment. However, our study should be limited by one cell line and one ER stressor. Further studies are required for the elucidation of the more detailed pathogenesis of hearing impairment by using other auditory cell lines and ER stress-inducing reagents.

## 4. Materials and Methods

### 4.1. Reagents and Antibodies

Tunicamycin was purchased from Sigma-Aldrich (St. Louis, MO, USA). Necrostatin-1 (Nec-1) was from Cayman Chemical (Ann Arbor, MI, USA). Z-Val-Ala-Asp-fluoromethylketone (zVAD-fmk) was from MBL (Nagoya, Japan). Necrosulfonamide (NSA) was from MedChem Express (Princeton, NJ, USA). The following primary antibodies were purchased from Cell Signaling Technology (Danvers, MA, USA): Anti-IRE1-α, anti-XBP1s, anti-RIPK1, anti-RIPK3, anti-MLKL, anti-caspase-8, anti-caspase-9, and anti-β-actin antibodies. Anti-goat mouse IgG and anti-rabbit IgG antibodies were from Santa Cruz Biotechnology, Inc. (Dallas, TX, USA) and Cell Signaling Technology, respectively. Small interfering RNA (siRNA) for control siRNA, caspase-8 siRNA, and caspase-9 were purchased from Santa Cruz Biotechnology, Inc., and RIPK3 siRNA were purchased from Cell Signaling Technology.

### 4.2. Cell Culture and Culture Condition

The HEI-OC1 (House Ear Institute-Organ of Corti 1) cell line was kindly provided by F. Kalinec (UCLA, Los Angeles, CA, USA) [58]. This conditionally immortalized mouse auditory cell line is a well-established in vitro system for investigating cellular and molecular mechanisms. The cells were cultured with high-glucose Dulbecco’s Modified Eagle’s Medium (DMEM; Gibco, Grand Island, NY, USA) containing 10% fetal bovine serum (FBS; Invitrogen, Carlsbad, CA, USA) and 1% penicillin-streptomycin (Gibco) at 33 °C in a humidified incubator with 10% CO2.

### 4.3. Cell Viability Assay

HEI-OC1 cells (5 × 10^4^ cells/well in 24 well plates) were incubated with 50 µg/mL of tunicamycin at 33 °C for designated periods of time. The viability was measured by staining with 0.4% trypan blue (Gibco) and manual counting with hemocytometer. The percentage of viability was defined as percent live per total cells.

### 4.4. Western Blot Analysis

The cells were seeded in 35 mm culture dishes at a density of 2 × 10^5^ cells/well and incubated with 50 µg/mL of tunicamycin for designated periods of time. After the designated treatment, cells were lysed in 300 µL of sodium dodecyl sulfate (SDS) buffer. The samples (10 µL) were subjected to electrophoresis on 4–12% or 12% sodium dodecyl sulfate-polyacrylamide gels (SDS-PAGE; Invitrogen) for 90–120 min at 20 mA and then transferred onto polyvinylidene difluoride (PVDF) membranes (Invitrogen) using iBlot (Life Technologies, Carlsbad, CA, USA). The membranes were blocked in Super Block Blocking Buffer in TBS (Thermo Fisher Scientific, Waltham, MA, USA) for 1 h and incubated overnight at 4 °C in the presence of primary antibodies at dilutions of 1:1000–1:3000 in Tris Buffered Saline with Tween 20 (TBS-T). After three washes with TBS-T, the membranes were incubated with the corresponding species-appropriate secondary antibodies at dilutions of 1:1000–1:3000 in TBS-T for 1 h. Then, the immunoreactive bands on the membrane were visualized with ECL Prime Western Blotting Detection Reagents (GE healthcare, Chicago, IL, USA) and the images were captured using the LAS-4000 mini (FUJIFILM, Tokyo, Japan). The band density was analyzed using Multi Gauge version 3.1 (FUJIFILM). The quantitative densitometric values for each protein were normalized to β-actin.

### 4.5. Co-Immunoprecipitation

Co-immunoprecipitation was performed using the Dynabeads^®^ Protein G Immunoprecipitation Kit (Thermo Fisher Scientific), according to the manufacturer’s instructions. The cells were seeded in 35 mm culture dishes at a density of 2 × 10^5^ cells/well and were incubated with 50 µg/ml tunicamycin for 24 h. The cells were lysed in RIPA buffer (Wako, Osaka, Japan) containing a protease inhibitor (Nacalai, Tokyo, Japan). Lysates were incubated overnight at 4 °C with the primary antibody, and then the beads were added and incubated for 90 min. After 3 washes using the washing buffer, the immunoprecipitates were eluted with 20 µL of SDS sample buffer by boiling for 10 min at 70 °C and were subsequently subjected to Western blot analysis.

### 4.6. Transmission Electron Microscopy

The cells were harvested by trypsinization, and then were fixed with 2.5% glutaraldehyde in 0.1 M cacodylic buffer solution (pH 7.4) overnight followed by 1% osmium tetroxide in 0.1 M cacodylic buffer solution (pH 7.4) for 2 h. Then, the samples were dehydrated through graded ethanol, and embedded in Quetol-812. Ultrathin sections were cut with a diamond knife on an ultramicrotome (ULTRACUT UCT, Leica, Wien, Austria), and were stained with uranyl acetate and lead citrate. The sections were observed using a transmission electron microscope (JEM-1200EX, JEOL, Tokyo, Japan).

### 4.7. Flow Cytometry Analysis

The Annexin V-FITC/Propidium iodide (PI) flow cytometric assay was used to detect cell death. Briefly, the cells were seeded in 60 mm culture dishes at a density of 6 × 10^5^ cells/well. The cells were incubated with 50 µg/mL of tunicamycin for 24 h. After removing the culture medium, the cells were washed with binding buffer, and then 5 µL of Annexin V-FITC and PI were added. The cells were immediately acquired by flow cytometer (GalliosFlowCytometer, Beckman Coulter, Brea, CA, USA) and analyzed using FlowJo software (FlowJo, LLC, Ashland, OR, USA). Cells that were PI negative and Annexin V negative were considered healthy, cells that were PI negative and Annexin V positive were considered early apoptotic, and cells that were positive to both PI and Annexin V were considered late apoptotic and necrotic.

### 4.8. Transient Small Interfering RNA (siRNA) Transfection

Small interfering RNA (siRNA) against RIPK3 (Cell Signaling Technology), caspase-8, caspase-9, and control siRNA (Santa Cruz Biotechnology, Inc.) were used for knockdown of the RIPK3, caspase-8, and caspase-9 genes. The cells were seeded in 35 mm culture dishes at a density of 2 × 10^5^ cells/well and cultured overnight to achieve 60–80% confluency. Then, the cells were transfected with siRNA using RNAiMAX reagent (Invitrogen), according to the manufacturer’s protocol. After 48 h of transfection, the cells were treated as designed.

### 4.9. Statistical Analysis

The experiments were performed independently, at least three times. The data were expressed as mean ± S.D. The differences between groups were determined by one-way analysis of variance (ANOVA), followed by the Bonferroni post hoc test, when appropriate. For analysis of the statistical analysis differences between two groups, an unpaired Student’s *t*-test was applied. 

## Supplementary Materials

Supplementary materials can be found at https://www.mdpi.com/1422-0067/20/23/5896/s1.
