# Supplementary material for: Caspase-8 Regulates Endoplasmic Reticulum Stress-Induced Necroptosis Independent of the Apoptosis Pathway in Auditory Cells
Source: Int J Mol Sci. 2019 Nov 24;20(23):5896. doi: 10.3390/ijms20235896 (PMC6928907; doi:10.3390/ijms20235896)

Supplementary Information

Caspase-8 regulates endoplasmic reticulum stress-induced necroptosis independent of the apoptosis pathway in auditory cells

Akihiro Kishino[1*], Ken Hayashi[2], Miyoko Maeda[1], Toyoharu Jike[3], Chiaki Hidai[4], Yasuyuki Nomura[1] and Takeshi Oshima[1]

[1] Department of Otolaryngology, School of Medicine, Nihon University, Tokyo 173-8610, Japan

[2] Department of Otolaryngology, Kamio Memorial Hospital, Tokyo 101-0063, Japan

[3] Research Institute of Medical Science, School of Medicine, Nihon University, Tokyo 173-8610, Japan

[4] Department of Physiology, School of Medicine, Nihon University, Tokyo 173-8610, Japan

*Corresponding author: Dr. Akihiro Kishino, Department of Otolaryngology, School of Medicine, Nihon University, Postal address: 30-1, Oyaguchi Kami-cho, Itabashi-ku, Tokyo, Japan 173-8610, Phone: +81-3-3972-8111, Fax: +81-3-3972-1321, E-mail: akkishino187@gmail.com

Supplementary Figure S1a

Representative transmission electron microscopy photomicrographs of low magnifying scales in HEI-OC1 cells treated with tunicamycin (50 μg/ml for 24 h). Tunicamycin-treated cells showed morphological characteristics of apoptosis (arrow) or necroptosis (arrowhead) (left and middle panel: 2,000×, and right panel: 2,500× magnifications).

Supplementary Figures S1-6.

Uncropped version of blots displayed in main figure 1f (Figures S1 b–f), 2c (Figures S2 a and b), 2f (Figures S2 c–e), 3b (Figures S3 a–d), 4a (Figures S4 a–d), 4d (Figures S4 e–i), 5a (Figures S5 a–c) and 5c (Figures S5 d–h).

Supplementary Figure S4j.

Representative western blots showing the expressions of caspase-8 and MLKL in tunicamycin-treated caspase-8 KD cells (50 μg/ml for 48 h). β-actin was included as the loading control. The expressions of caspase-8 and MLKL were detected, and the means ± S.D. (fold changes compared to the control group) of three or more independent studies were presented.

Supplementary Figure 1.

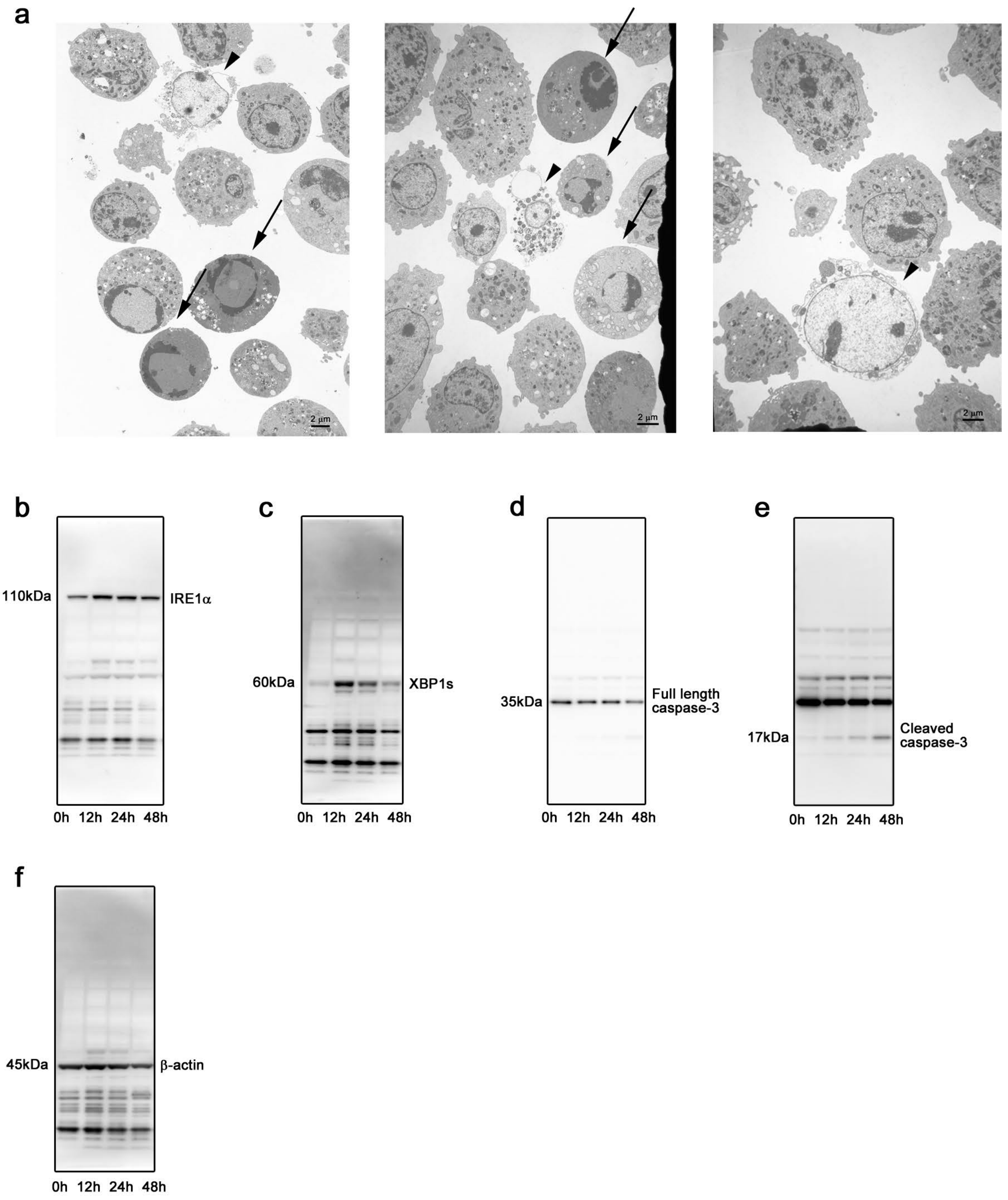

Supplementary Figure 2.

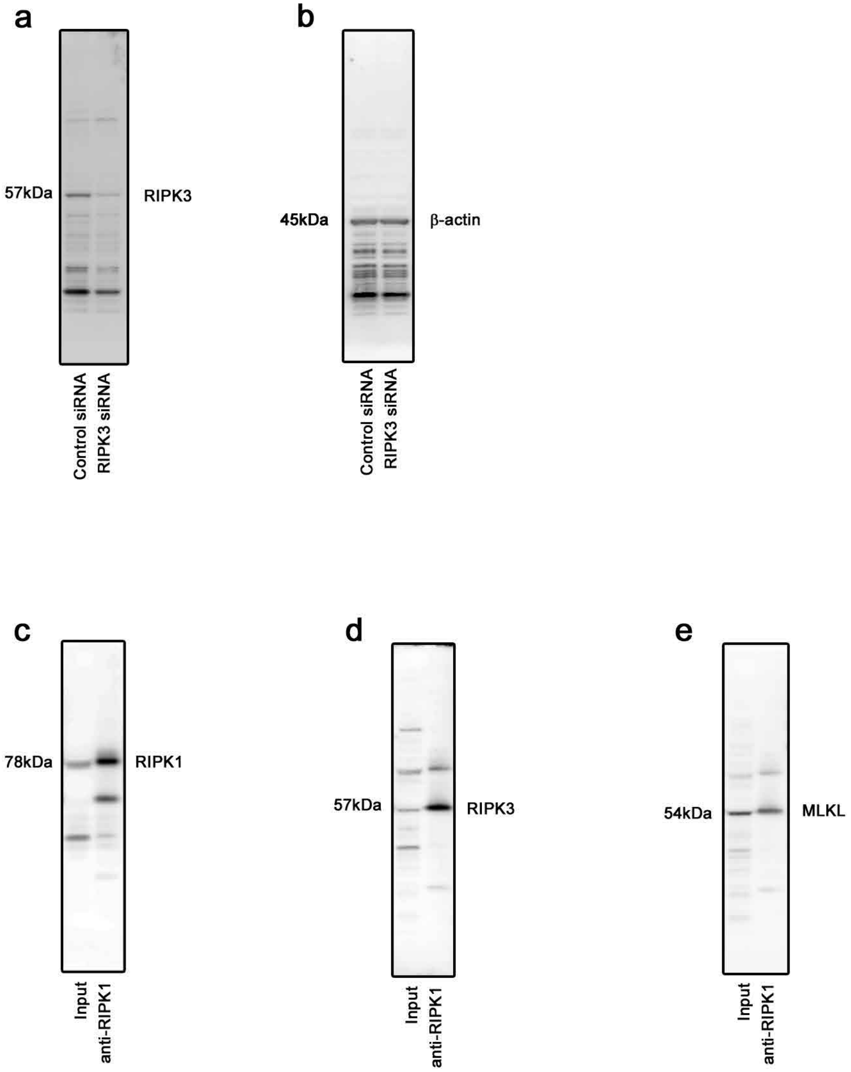

Supplementary Figure 3.

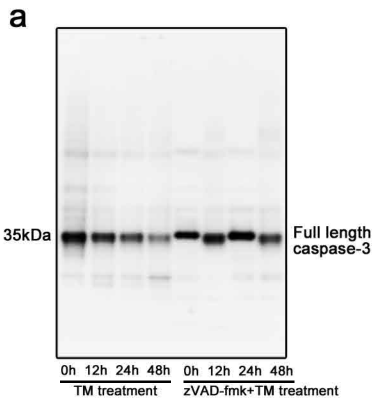

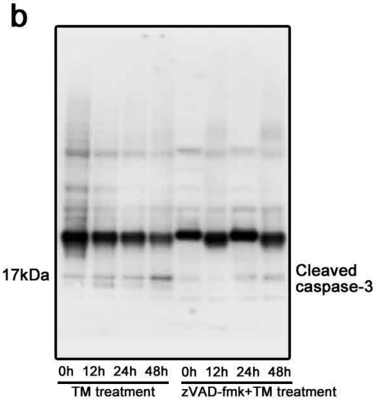

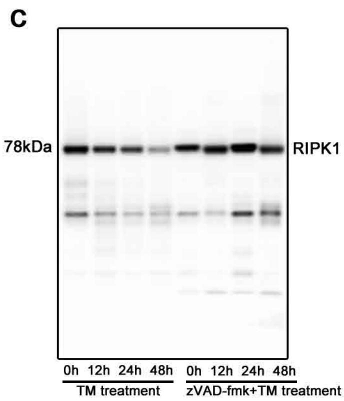

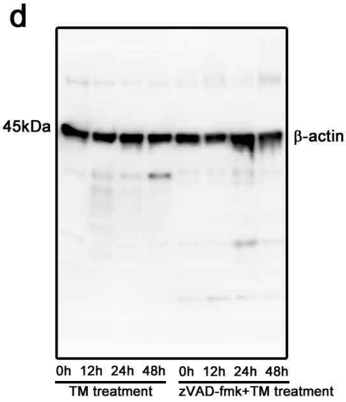

Supplementary Figure 4.

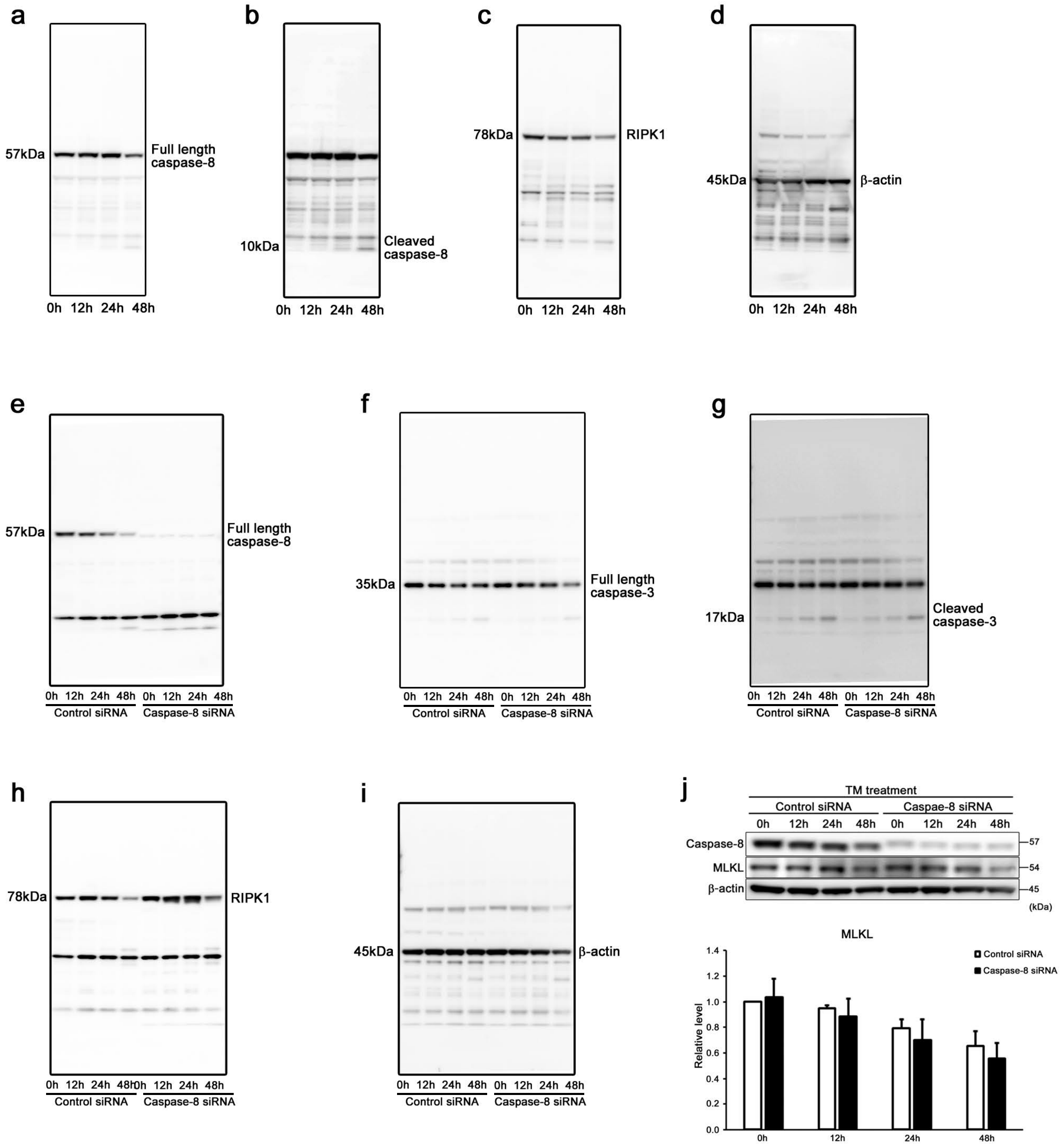

Supplementary Figure 5.

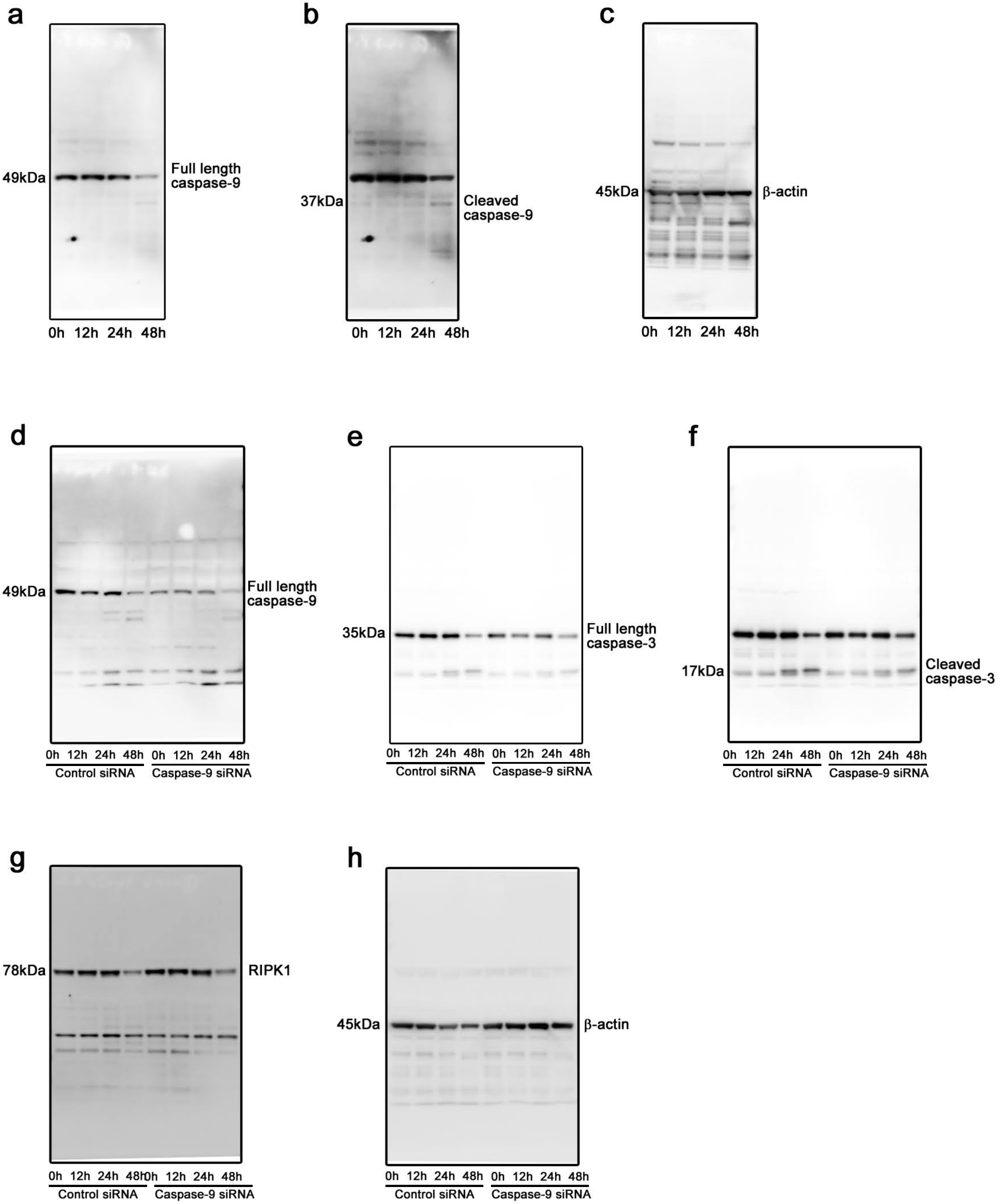

Supplement: Supplementary file 1 [file ijms-20-05896-s001.pdf]
